# An unusual presentation of neuroglial heterotopia: case report

**DOI:** 10.1259/bjrcr.20190116

**Published:** 2020-09-29

**Authors:** Parthasarathy Karunakaran, Chary Duraikannu, Venkata Narasimha Kumar Pulupula

**Affiliations:** 1 Department of Radiology, Countess of Chester NHS foundation trust, Chester, United Kingdom

## Abstract

We report a rare case of nasopharyngeal neuroglial heterotopia in a 16-year-old girl who presented with sore throat and feeling of a lump in her throat. Neuroglial heterotopia is a mass composed of misplaced neural tissue during embryonic development which has lost its intracranial connection. A careful review of literature in PUBMED shows most of the previously reported cases of nasopharyngeal glial heterotopia presented during neonatal or infancy period with symptoms of respiratory distress or airway obstruction. Our case caused a diagnostic dilemma due to late presentation and atypical radiological findings. Imaging, especially MRI, is vital for evaluating such nasopharyngeal masses in children for pre-surgical planning and more importantly to rule out any intracranial communication. Treatment is surgical resection by endoscopic or external approach, with a rare possibility of recurrence.

## Introduction

Neuroglial heterotopia, commonly termed nasal glioma is a non-hereditary abnormality of misplaced glial tissue in which intracranial continuity has become obliterated.^1^ The term nasal glioma is a misnomer, as it is not a true neoplasm. The usual presentation is a mass around the nose presenting with respiratory distress in infancy or early childhood.^2^ It is an extremely rare benign developmental malformation and commonly misdiagnosed as encephalocele, teratomas, or hemangioma.^3^ Preoperative imaging with MRI is crucial in characterizing these lesions, determining its extent and to exclude any intracranial communication. Herein, we present a case of neuroglial heterotopia in the nasopharynx with late onset presentation and atypical imaging characteristics.

## Case report

A 16-year-old female presented to ENT clinic with 4 weeks history of sore throat and feeling of a lump in her throat for 2 weeks, associated with nasal voice. There was no difficulty in swallowing or any constitutional symptoms. She had visited the GP earlier and was treated with antibiotics for tonsillitis. On examination, palate and tonsils were normal. Further assessment with a nasal endoscopy revealed a grape-sized vascular appearing lesion in the post-nasal space. She was later referred for MRI neck to characterize the lesion.

**Figure 1.**
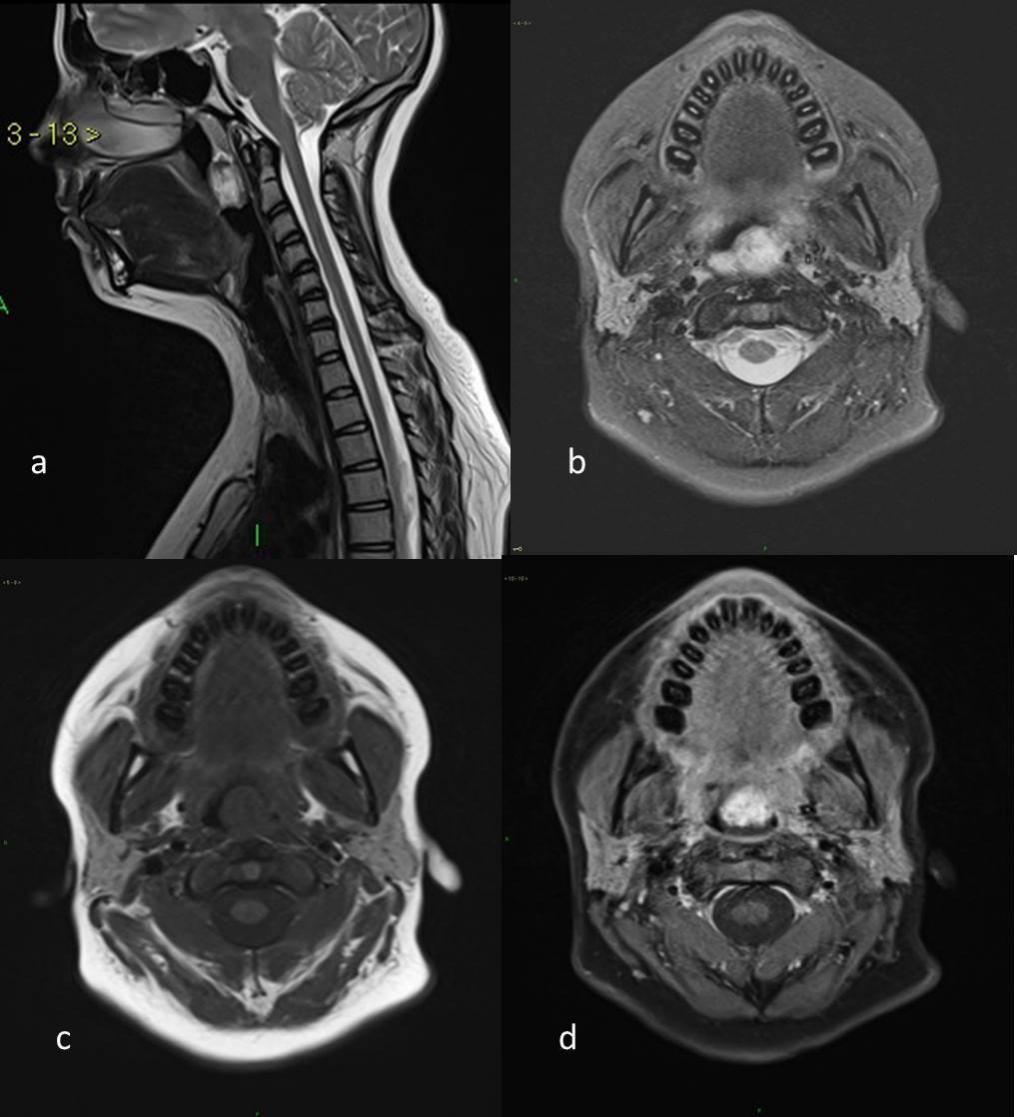
Sagittal T2 (a) and axial T2FS (b) MR images show heterogeneous hyperintense lesion in nasopharynx causing mild airway narrowing. The lesion appears isointense in ﻿﻿T1 WI (c) and enhances in post contrast T1 weighted FS sequence (d). FS, fat suppressed; T1 WI, T1 weighted imaging.

MRI revealed a well-circumscribed, ovoid soft tissue intensity lesion measuring 2.8 × 1.8 × 1.5 cm in the mid-line of posterior nasopharyngeal wall, corresponding to C2 vertebral level. The lesion showed isointense signal in T1 weighted imaging, heterogeneously hyperintense signal in T2 weighted imaging and moderate enhancement post-intravenous gadolinium contrast (Figure 1). The lesion caused mild anterior displacement of the uvula and narrowing of nasopharyngeal airway. There was no intracranial communication to the lesion.

**Figure 2.**
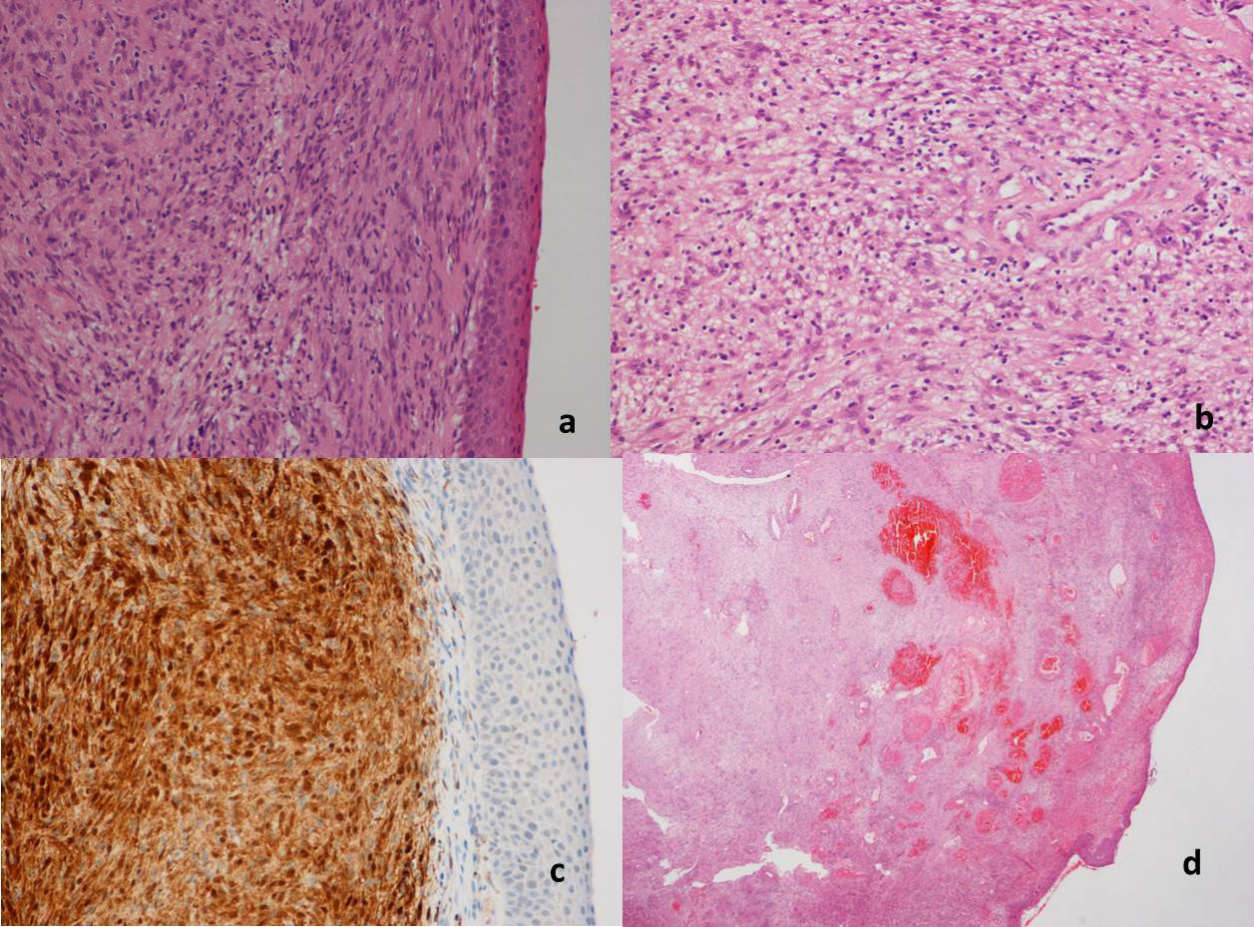
Histology revealed neuroglial tissue with overlying transitional epithelium (a), glial tissue seen under magnification (b) which stained positively for S-100 (c). There is ulceration and hypervascularity of the overlying epithelium (d).

These imaging findings and clinical presentation indicated a provisional diagnosis of a hemangioma. Later, the patient underwent complete surgical excision of the lesion via transnasal endoscopic approach. Histological examination revealed glial heterotopia with ulceration and hypervascularity of the overlying epithelium. On further immunohistochemistry, the neural elements stained positively for S-100 protein (Figure 2).

## Discussion

Neuroglial heterotopias are developmental midline masses of neurogenic origin and are characterized by the presence of dysplastic non-teratomatous extracranial glial tissue losing its intracranial communication.^4^ They commonly occur around the nose and nasopharynx, not necessarily in the midline. They are rare and occur in about 20,000–40,000 births.^5^ Over 181 cases of neuroglial heterotopia have been documented in the literature with 98% presenting in early infancy.^6^ A careful review of literature in PUBMED during 1990–2018 revealed about 14 cases of glial heterotopias reported in the nasopharyngeal region, and all presented during neonatal or infancy period with symptoms of respiratory distress or airway obstruction. Extra nasal sites are rare and include the orbit, palate, submandibular region, and overlying the spine.^7–9^ Nasal gliomas can occur in association with other anomalies, which include cleft palate, micrognathia, Pierre Robin syndrome and congenital heart defects.^10^

Clinical presentation of nasopharyngeal heterotopias is usually with respiratory distress, neck mass and feeding difficulties.^6^ In our case, although the lesion is situated in the nasopharynx, the delayed presentation is presumably due to its small size.

MR signal characteristics in neuroglial heterotopia are variable depending on its contents. They may be heterogeneously T2 hyperintense due to the presence of dysplastic cells^11^ and/or contain cystic components due to functioning choroid elements with cerebrospinal fluid production. No cystic areas were identified in our case and intense enhancement is likely due to the hypervascularity of the overlying epithelium which was noted histologically. Although CT can complement in assessing bony defects, it was not performed in our case, as there was no evidence of intracranial extension.

Histologically, these lesions are composed of a variety of elements including astrocytes, oligodendrocytes, neurons, functioning choroid plexus and retinal cells interspersed in fibrous stromal tissue.^8^ In case of significant background fibrosis, the glial cells could be identified by glial fibrillary acidic protein and S-100 protein immunohistochemistry.^12^

They are treated by complete surgical excision either via trans-oral or endoscopic approach depending on the location of the mass. Recurrence is rare and can be seen in cases with an intracranial connection.

Differentials for nasopharyngeal lesions presenting in early childhood would include encephalocele, hemangiomas, dermoid and rhabdomyosarcoma. Considering the late onset of presentation and atypical enhancement features, our case is distinct from the ones described in the literature. For the reasons cited, hemangioma, and not neuroglial heterotopia, was our prime consideration. There was no fat or cystic component and no intracranial connection, which helped to exclude other differentials.

## Conclusion

Radiological assessment of midline neck masses is best performed with MRI. Although histopathology remains the gold-standard in atypical presentation, imaging is crucial in characterizing these lesions and to determine their extent. This is therefore helpful in proper surgical planning and to prevent recurrence. The possible existence of intracranial continuity must be ruled out preoperatively, differentiating neuroglial heterotopia from an encephalocele. Early detection and management are crucial to relieve obstructive symptoms and maintain normal development.

## Learning points

- Neuroglial heterotopias, although rare, is an important differential for intranasal and nasopharyngeal masses in pediatric population.
- Preoperative imaging with MRI is crucial for nasopharyngeal masses in determining the extent, assess the vascularity and to exclude any intracranial communication.
